# OsSGL, a Novel DUF1645 Domain-Containing Protein, Confers Enhanced Drought Tolerance in Transgenic Rice and *Arabidopsis*

**DOI:** 10.3389/fpls.2016.02001

**Published:** 2016-12-27

**Authors:** Yanchun Cui, Manling Wang, Huina Zhou, Mingjuan Li, Lifang Huang, Xuming Yin, Guoqiang Zhao, Fucheng Lin, Xinjie Xia, Guoyun Xu

**Affiliations:** ^1^Key Laboratory of Agro-ecological Processes in Subtropical Region, Institute of Subtropical Agriculture, Chinese Academy of SciencesChangsha, China; ^2^Zhengzhou Tobacco Research Institute of China National Tobacco CorporationZhengzhou, China; ^3^Postdoctoral Research Stations of Basic Medical Science, Zhengzhou UniversityZhengzhou, China; ^4^State Key Laboratory for Rice Biology, Biotechnology Institute, Zhejiang UniversityHangzhou, China

**Keywords:** *OsSGL*, drought tolerance, rice, DUF1645 domain, root, overexpression

## Abstract

Drought is a major environmental factor that limits plant growth and crop productivity. Genetic engineering is an effective approach to improve drought tolerance in various crops, including rice (*Oryza sativa*). Functional characterization of relevant genes is a prerequisite when identifying candidates for such improvements. We investigated OsSGL (*Oryza sativa* Stress tolerance and Grain Length), a novel DUF1645 domain-containing protein from rice. *OsSGL* was up-regulated by multiple stresses and localized to the nucleus. Transgenic plants over-expressing or hetero-expressing *OsSGL* conferred significantly improved drought tolerance in transgenic rice and *Arabidopsis thaliana*, respectively. The overexpressing plants accumulated higher levels of proline and soluble sugars but lower malondialdehyde (MDA) contents under osmotic stress. Our RNA-sequencing data demonstrated that several stress-responsive genes were significantly altered in transgenic rice plants. We unexpectedly observed that those overexpressing rice plants also had extensive root systems, perhaps due to the altered transcript levels of auxin- and cytokinin-associated genes. These results suggest that the mechanism by which *OsSGL* confers enhanced drought tolerance is due to the modulated expression of stress-responsive genes, higher accumulations of osmolytes, and enlarged root systems.

## Introduction

Rice (*Oryza sativa*) is the most important food sources for more than half of the world’s population. However, water is already a scarce resource in many parts of the world, and increasingly frequent drought events present enormous challenges for sustainable rice production (Zhang, 2007). Drought is a major environmental factor that adversely affects plant growth and development, thus limiting agricultural productivity. Under stress conditions, plants can initiate a range of alterations at the molecular, cellular, and physiological levels, including stomata closure, reduced photosynthesis, higher accumulations of osmolytes, and the induction of many stress-responsive genes (Shinozaki and Yamaguchi-Shinozaki, 2007). Genetic engineering is considered an alternative for enhancing stress tolerance, and has greatly contributed to agronomic trait modifications in crop species. Many genes encoding functional proteins, transcription factors, and proteins involved in signaling pathways have been identified as abiotic stress-responsive genes (Shinozaki and Yamaguchi-Shinozaki, 2007; Masclaux-Daubresse et al., 2010; Turan et al., 2012). Tolerant plants have been engineered through heterologous expression of genes that encode functional or regulatory proteins (Golldack et al., 2011; Kim M. et al., 2013; Lu et al., 2013; Li et al., 2014).

Root morphology is one of the most promising traits for improving stress tolerance. An ideal root architecture and extensive root system can maximize water capture and allow access to moisture at the most suitable soil depth, thereby supporting shoot growth under drought conditions (Asch et al., 2005; Lynch, 2013). Genotypes with deeper roots generally have greater yields than shallow-rooted ones during drought periods (Ho et al., 2005; Hund et al., 2009; Lopes and Reynolds, 2010; Henry et al., 2011) and deeper rooting is more prevalent among species that grow in water-deficient environments (Schenk and Jackson, 2005). Some rice varieties with well-developed root systems have an advantage in grain yields under stress conditions (Price et al., 1997; Serraj et al., 2009). This improved yield potential can translate into better performance under stress scenarios but can also place a greater demand on water and other resources. However, root-specific expression of *OsNAC5* and *OsNAC9* in rice allows for the production of transgenic plants with an altered root architecture and enhanced drought tolerance without any yield penalty (Redillas et al., 2012; Jeong et al., 2013). Nelson et al. (2007) identified a transcription factor in Arabidopsis, AtNF-YB1, which confers drought tolerance. Transgenic maize (*Zea mays*) that constitutively expresses the orthologous gene *ZmNF-YB2* is drought-tolerant and has improved yields under water-limited conditions. In addition, overexpression of *DEEPER ROOTING 1* in rice confers the capacity for drought avoidance by altering root architecture (Uga et al., 2013). Overexpression of that gene results in a drought-related accumulation of lignin in those roots, which modifies cell wall architecture and enhances growth under stress conditions (Yoshimura et al., 2008).

Despite the tremendous progress made in determining gene functions in rice, many genes have not yet been characterized at the biochemistry and biological function levels. In eukaryotes, Domains of Unknown Function (DUF) proteins are part of several gene families that encode functionally uncharacterized proteins. A naming scheme for DUFs was established when DUF1 and DUF2 were added into the SMART database in the 1990s (Schultz et al., 1998). Those proteins have been categorized and enumerated as DUFX, where X represents the order of addition to the Protein-family (Pfam) database (Bateman et al., 2010). That database now contains over 3000 DUF families, and members within a particular family share conserved domains in their coding region (Bateman et al., 2010; Punta et al., 2012). Different DUF families play various roles in plant development and stress responses. *Arabidopsis ESK1*, a member of the DUF231 domain proteins, is a novel negative regulator of cold acclimation (Xin et al., 2007) while two other members, *TBR* and *TBL3*, are involved in cellulose synthesis and deposition of the secondary cell wall (Bischoff et al., 2010). The DUF640 domain-like gene *TH1* is mainly expressed in young inflorescences of rice as well as in the spikelet lemmas and paleas, where it regulates their development (Li et al., 2012). Two Arabidopsis DUF1117 E3 ubiquitin ligase genes, *AtRDUF1* and *AtRDUF2*, are induced by drought and ABA, and both single- and double-knockout mutants exhibit reduced tolerance to ABA-mediated drought stress (Kim et al., 2012). These studies indicate that DUF proteins play important roles in plant growth and stress adaptations. However, little is known about the molecular mechanisms by which those proteins confer drought tolerance.

To decipher the relevant genes in rice, we analyzed the genome expression profiles of rice Pei’ai 64S (*Oryza sativa* L.) under different stresses by rice microarray (unpublished data). One such gene, *OsSGL*, on Chromosome 2, encodes a putative DUF1645 domain-containing protein and lacks introns. Members of DUF1645 (PF07816) are derived from a number of hypothetical plant proteins. However, the gene family has not been characterized and no member has been functionally studied. Therefore, we investigated the role of *OsSGL* in the drought response. Transgenic rice plants over-expressing *OsSGL* showed enhanced tolerance during the seedling and vegetative stages, with altered transcript levels of stress-responsive genes, an enlarged root system, and higher osmolyte content. In addition, transgenic Arabidopsis that hetero-expressed this gene were more tolerant to osmotic stress.

## Materials and Methods

### Plant Materials and Growth Conditions

Rice seeds (*O. sativa* L. ssp. *Indica* cv 9311) were surface-sterilized with 75% ethanol for 1–2 min, followed by 50% sodium hypochlorite for 25 min and washed with distilled water at least three times. For rice root measurement, sterilized seeds were germinated on ½12-strength Murashige and Skoog (MS) (Murashige and Skoog, 1962) for 7 days at 28°C under the photoperiod of a 12 h light and 12 h dark photoperiod. *Arabidopsis* (*A. thaliana* ecotype Columbia (Col-0) was used as wild-type (WT) in this study. Seeds were sterilized with 75% ethanol and 10% bleach (v/v), followed by rinsing with sterile water for at least three times, and then vernalized for 2 days at 4°C. Plants were grown on ½12 MS medium containing 0.8% (w/v) sucrose and 0.75% (w/v) agar in a growth chamber maintained at 22°C and 60% relative humidity under the photoperiod of a 16 h light and 8 h darkness photoperiod.

### Plasmid Construction and Transformation

The coding region of *OsSGL* was amplified from total RNA of rice Pei’ ai 64S, using an RT-PCR system (Promega, Madison, WI, USA) according to the manufacturer’s instructions. The verified PCR fragment was digested and ligated into binary expression vector p1300-pJITl63, which was derived from pCAMBIA1300 and pJITl63, carrying the constitutive cauliflower mosaic virus (CaMV) 35S promoter. This yielded overexpression vector p1300-pJITl63-*OsSGL*. Thus, expression of *OsSGL* was controlled by the CaMV 35S promoter. All enzymes and reagents used for PCR or restriction digestion were purchased from Takara (Japan). Primer pairs used are listed in Supplementary Table S1.

For *Arabidopsis* transformation, binary construct p1300-pJITl63-*OsSGL* was introduced into *Agrobacterium tumefaciens* strain GV3101. Transformation was performed with the floral-dipping method (Clough and Bent, 1998). Transformants (T_1_) were selected on a ½12 MS medium supplemented with 40 mg L^-1^ hygromycin B. The T_3_ lines displaying 100% hygromycin resistance were considered homozygous and used for further experiments.

For rice transformation, plasmid p1300-pJITl63-*OsSGL* was introduced into *A. tumefaciens* strain EHA105, and embryogenic calli induced from rice cultivar 9311 (*O. sativa* L. ssp. *Indica*) which is widely used as a male parent in the production of high yield rice in china were used as the transformation receptor. Our method followed that described by Xiao et al. (2008). Hygromycin resistance was used to screen positive transgenic plants. Using primers specific for the hygromycin phosphotransferase gene, we performed PCR to confirm the presence of the T-DNA in the transformants. The expression level of *OsSGL* in positive transgenic plants was determined by semi-quantitative reverse-transcription PCR (RT-PCR) for Arabidopsis and quantitative real-time PCR (qPCR) for rice. *Tubulin* and *Actin2* were used as the internal control in *Arabidopsis* and rice, respectively. All primers are listed in Supplementary Table S1. We used T_3_ homozygous plants in subsequent experiments.

### Quantitative Real-Time PCR and Semi-quantitative RT-PCR

Total RNAs were extracted with TRIzol reagent (Invitrogen, Burlington, ON, Canada) according to the manufacturer’s instructions. Afterward, 1 μg of DNase-treated RNA was reverse-transcribed using a PrimeScript^TM^ 1st Strand cDNA synthesis kit (Takara) according to the manufacturer’s protocol. The qPCR analysis was conducted with FastStart Universal SYBR Green Master (Roche), and reactions were performed in an ABI 7900HT (Applied Biosystems) at 95°C for 10 min, followed by 40 cycles at 95°C for 15 s and 58°C for 30 s. The internal controls were *Tubulin* and *Actin2* for *Arabidopsis* and rice, respectively. Each sample was analyzed in triplicate and relative amounts of mRNA were calculated per the comparative threshold cycle method.

For semi-quantitative RT-PCR, two-step RT-PCR method was chosen. Total RNA extractions and cDNA synthesis were performed as described above. All PCR amplifications were conducted in a total volume of 20 μL under the following conditions: 22–27 cycles of denaturation (94°C, 30 s), annealing (58°C, 35 s), and extension (72°C, 30 s). The primer pairs for semi-quantitative RT-PCR and qPCR are listed in Supplementary Table S1.

### Subcellular Localization

To determine the subcellular localization of OsSGL in rice protoplasts, we amplified the full-length open reading frame (ORF) of *OsSGL* without the termination codon. After verification by sequencing, the PCR fragment was cloned into vector pJITL63-GFP to produce an OsSGL-GFP fusion construct (CaMV35S-*OsSGL*-*GFP*), thus allowing the fusion gene to be driven by the CaMV 35S promoter. Construct CaMV35S-*GFP* was used as the control. Transformation mediated by polyethylene glycol (PEG) was performed as described previously (Zhang et al., 2011). After 6–18 h of incubation in the dark, the subcellular location of the recombinant proteins was observed with a confocal laser scanning microscope (Leica TCS 5 STED CW SP5).

### Drought and Osmotic Treatment

For the analysis of osmotic tolerance in *Arabidopsis*, sterilized seeds of WT and *OsSGL* transgenic plants were sown in triplicate on ½12 MS medium containing different concentrations of mannitol (0 or 300 mM). After vernalization for 2 days at 4°C in the darkness, plates were transferred to a growth room (22°C, 16 h light/8 h dark) and kept for another 9 days. At the end of the treatment, survival rates were determined. Plants with green cotyledons were designated as survivors. For analyses of the effects of osmotic stress on root length, following vernalization, seeds of WT and transgenic plants were germinated on ½12 MS medium for 3 days. Plants with identical growth performance were transferred to ½12 MS medium containing 300 mM mannitol, and grown for another 12 days. Plates were placed vertically to facilitate comparison of root growth.

To determine drought tolerance in transgenic rice, T_3_ homozygous seeds were used for further analysis. Drought assays were performed in a controlled growth chamber PGC15.5 (Percival, Perry, IA, USA). For osmotic stress at the post-germination stage, sterilized seeds were sown on ½12 MS medium containing 0 or 400 mM mannitol for 11 d. Root and shoot were measured at the end of the treatments. For drought assays at seedling stage, WT and transgenic lines were planted in the same buckets. At the four-leaf-stage, watering was withheld and resumed 5 days later. After 3-week recovery, survival rates were determined.

### Morphological Analysis of Roots

To determine the root morphological parameters at seedling stage, seeds were germinated on ½12 MS medium and kept growing for 7 days in a growth room. Three replicates were performed. Lengths of primary, adventitious and lateral roots were measured. For the determination of adventitious root, the five longest adventitious roots on each seedling were counted. Similar to measurement of adventitious root length, lateral root length was determined with the 15 longest lateral roots on each primary root. For root comparison at the vegetative and reproductive stages, plants of WT and transgenic lines were planted in the same big bucket. After 6 or 12 weeks, roots were rinsed clean by running water, root lengths and dry weights were measured.

### Determining the Contents of Proline, Soluble Sugars, and Malondialdehyde (MDA)

Four-week-old rice seedlings were used for biochemical analysis. Both WT and transgenic plants were transferred from the basal nutrient solution to a nutrient solution supplemented with 20% PEG. After 2 days of treatment, proline and soluble sugar contents in harvested tissue samples were measured according to the sulphosalicylic acid method (Troll and Lindsley, 1955) and the anthrone method (Morris, 1948), respectively. The level of MDA was determined with thiobarbituric acid, as described by Kramer et al. (1991).

### Gene Expression Analysis by RNA-sequencing

RNA samples were sent to the Beijing Genomics Institute (BGI) for further RNA-seq analysis. Briefly, total RNA was isolated with TRIzol reagent from the aerial parts of 15-day-old transgenic and WT 9311 rice plants that treated with 20% PEG (w/v) for 6 h. Material from 10 plants of each genotype was pooled for RNA extraction, and sequencing was performed in an Illumina HiSeq 2000 sequencing platform (Beijing, China). Clean reads were mapped to the Rice Annotation Project Database (RAP-DB) using TopHat Version 2.0.9 (Kim D. et al., 2013). The abundance of mapped reads was normalized to RPKM (reads per kilobase of exon model per million mapped reads) (Mortazavi et al., 2008). The DEGseq package was used for identifying genes differentially expressed between sample pairings (i.e., WT vs. transgenic), and *P*-values were adjusted according to the Benjamini and Hochberg method. RNA-seq read data in this article has been deposited in the National Center for Biotechnology Information (NCBI) SRA database under the accession number SRP094588.

### Statistical Analysis

All statistical analyses were performed with Student’s *t*-tests using SPSS software version 13.0.

## Results

### Sequence and Phylogenetic Analyses of *OsSGL*

**FIGURE 1:**
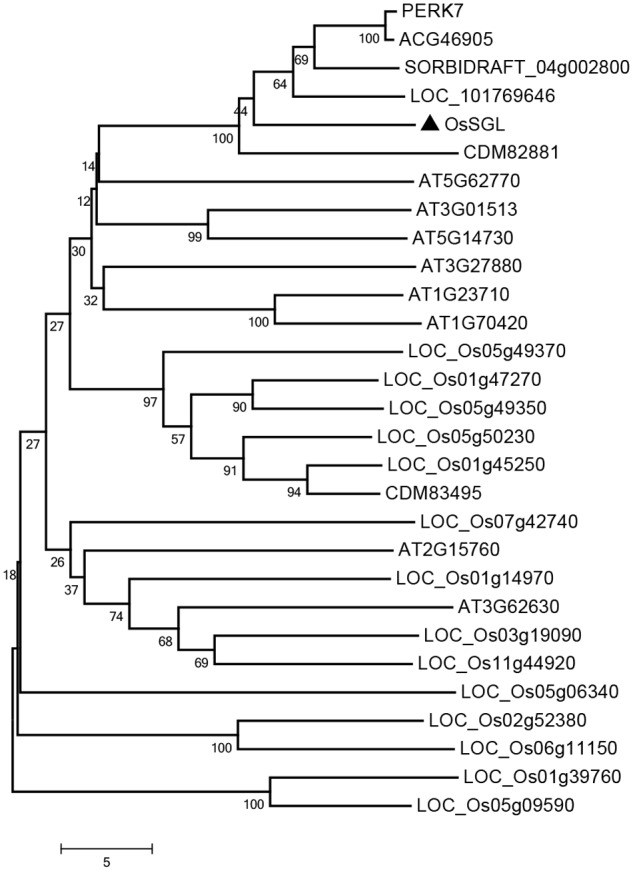
**Phylogenetic relationship of OsSGL with other DUF1645 proteins.** Multiple sequence alignment was performed via MEGA 6 and Neighbor-Joining method. Accession numbers include *Oryza sativa*: LOC_Os01g14970, LOC_Os01g39760, LOC_Os01g45250, LOC_Os01g47270, LOC_Os02g52380, LOC_Os03g19090, LOC_Os05g06340, LOC_Os05g09590, LOC_Os05g49350, LOC_Os05g49370, LOC_Os05g50230, LOC_Os06g11150, LOC_Os07g42740, and LOC_Os11g44920; *Arabidopsis thaliana*: AT1G23710, AT1G70420, AT2G15760, AT3G01513, AT3G27880, AT3G62630, AT5G14730, and AT5G62770; *Triticum aestivum*: *CDM82881* and *CDM83495*; *Zea mays*: ACG46905 and PERK7; *Setaria italica*: LOC_101769646; and *Sorghum bicolor*: SORBIDRAFT_04g002800.

The cDNA of *OsSGL* is 1062 bp long and contains an ORF of 768 bp flanked by a 5′-untranslated region (105 bp) and a 3′-untranslated region (189 bp). This gene encodes a novel protein of 255 amino acid residues with a molecular mass of 26.73 kDa. Bioinformatics analysis showed that OsSGL has a conserved domain of DUF1645 (Pfam PF07816) from amino acid residues 69-227^1^. To investigate the possible evolutionary relationships between OsSGL and other DUF1645 proteins, we constructed a phylogenetic tree via the Neighbor-Joining method, using full-length amino acid sequences (**Figure 1**). This protein is relatively closely related to its homologs in maize, *Triticum aestivum, Setaria italica*, and *Sorghum bicolor*. However, the relationship between OsSGL and other rice DUF1645 proteins is more divergent, suggesting that they play different roles in rice.

### Expression Pattern of *OsSGL* and Its Subcellular Localization

**FIGURE 2:**
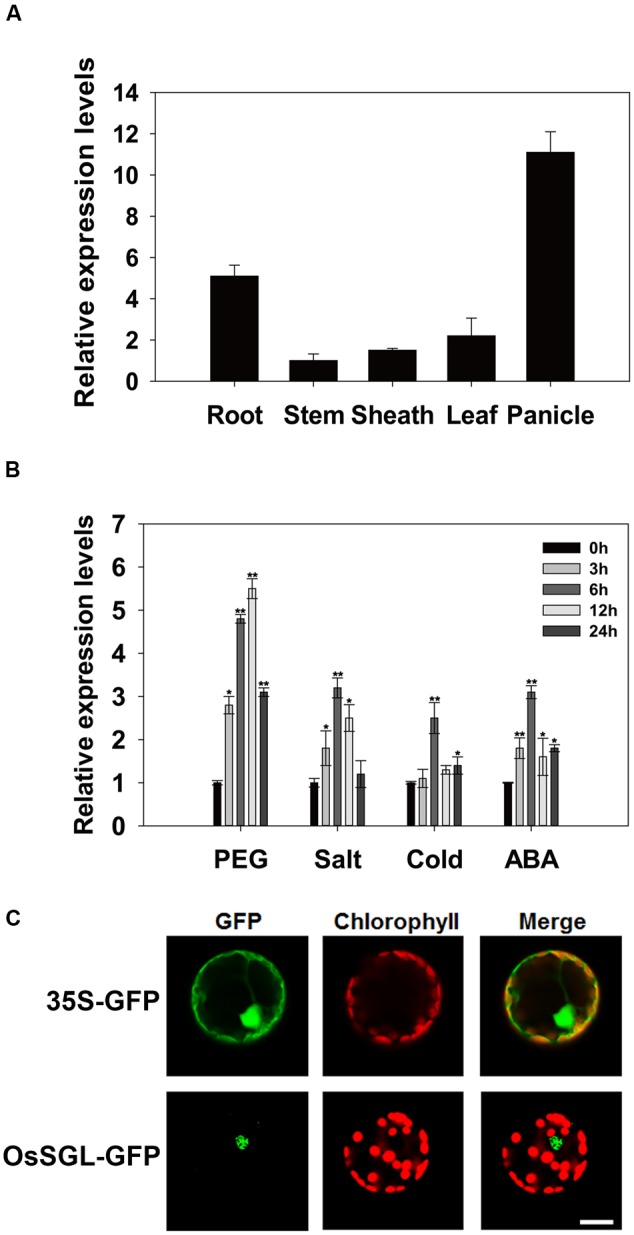
**Expression pattern and subcellular localization of *OsSGL*. (A)** Expression profile of *OsSGL* in organs at different developmental stages. **(B)** Relative expression of *OsSGL* under drought, salt, cold, or ABA stress. Materials were harvested at indicated time intervals for qPCR. Error bars represent SE for 3 independent experiments. **(C)** Subcellular localization of GFP control (upper panel) and OsSGL-GFP fusion protein (lower panel) in rice protoplast cells. CK, controls; Scale bars = 10 μm. ^∗^ and ^∗∗^ indicate significant difference from WT at *P* < 0.05 and < 0.01, respectively, by Student’s *t*-tests.

To elucidate the physiological and functional relevance of *OsSGL*, we examined its expression profile in young roots, mature stems, panicles at the heading stage, mature leaf blades, and mature leaf sheaths. Our qPCR results revealed that this gene was constitutively expressed in almost all tissues examined, with expression being greatest in the roots and panicles (**Figure 2A**). We also performed qPCR analysis using 2-week-old rice seedlings exposed to osmotic, salt, or cold stress and found that expression was highly up-regulated by all three as well as by ABA treatment (**Figure 2B**).

Subcellular localization of OsSGL was determined using a gene construct containing a DNA fragment that encodes OsSGL-GFP fusion protein driven by the CaMV35S promoter. Transient expression of *OsSGL* was analyzed by confocal laser scanning microscopy. As depicted in **Figure 2C**, cells expressing the control GFP gene showed cytoplasmic and nuclear distribution of the GFP signals. In contrast, cells expressing the OsSGL-GFP fusion gene showed GFP signal only in the nucleus, suggesting that OsSGL is a nucleus-localized protein.

### Rice and *Arabidopsis* Plants Over- or Hetero-Expressing *OsSGL* Have Enhanced Drought or Osmotic Tolerance

**FIGURE 3:**
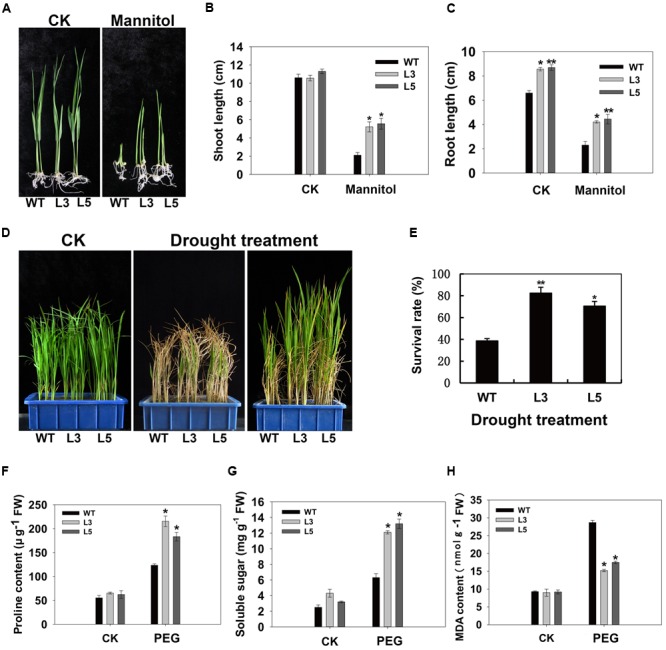
**Osmotic and drought tolerances in *OsSGL* transgenic plants. (A)** Phenotypic comparison of WT and transgenic plants under normal and osmotic stress conditions at post-germination stage. Shoot **(B)** and root **(C)** lengths were measured after 11 days of mannitol treatment. **(D)** *OsSGL* improves drought tolerance in transgenic rice. Water was withheld from four-leaf-stage seedlings (Left panel) and irrigation resumed 5 d later. After 3 weeks of recovery (right panel), survival rates were determined. Middle panel represents 1 week of recovery after re-watering. **(E)** Survival rates of wild type (WT) and *OsSGL* transgenic rice plants after drought treatment. **(F)** Comparison of the contents of proline in WT and *OsSGL* transgenic plants. **(G)** Measurement of soluble sugars in WT and *OsSGL* transgenic plants. **(H)** Comparison of MDA content in WT and *OsSGL* transgenic plants. For the measurement of proline, soluble sugars and MDA contents, 4-week-old WT and transgenic plants were treated with 20% PEG for 2 d before measuring. Data are means ± SE (*n* = 3). ^∗^ and ^∗∗^ indicate significant difference from WT at *P* < 0.05 and < 0.01, respectively, by Student’s *t*-tests. CK, controls; WT, 9311; L3 and L5, transgenic lines L3 and L5, respectively.

To examine the biological function of *OsSGL*, we generated transgenic rice and *Arabidopsis* plants with a vector containing its full-length ORF, driven by the CaMV 35S promoter. As we had expected, this gene was variably expressed in the transgenic lines. For further phenotypic analysis, we selected homozygous T_3_ transgenic *Arabidopsis* lines B2 and B4 and rice lines L3 and L5, all of which demonstrated higher levels of expression (Supplementary Figures S1 and S2). Strong induction of *OsSGL* expression by stresses suggested that the gene might be involved in stress tolerance. No obvious difference was observed in shoots between transgenic rice lines and WT under normal condition, while transgenic rice lines exhibited longer shoots than the WT under osmotic condition at the post-germination stage. In contrast, roots from transgenic lines were longer than the WT 9311 roots under both normal and osmotic conditions (**Figures 3A–C**). To assess drought tolerance at the seedling stage, we withheld irrigation for a short period before returning to a normal watering regimen. By Day 5 of this simulated-drought treatment, all WT plants were severely affected whereas the transgenic lines showed less leaf rolling and wilting. Following the recovery period, the transgenic plants also grew more vigorously (**Figure 3D**). After recovery, only 37.9% of the WT plants still contained green tissues while an average of 78.7% of the transgenic rice had survived the drought stress (**Figure 3E**).

The possible physiological mechanism responsible for improved drought tolerance was investigated by noting changes in stress-relevant parameters, i.e., proline, soluble sugar, and MDA contents. Under normal growing conditions, proline and MDA contents did not differ significantly between WT and *OsSGL* transgenic rice plants, but the level of soluble sugars was slightly higher in the latter. Upon exposure to osmotic stress, proline and soluble sugar contents were significantly elevated in all genotypes, but those increases were much more evident in the transgenic lines than in the WT (**Figures 3F,G**). Moreover, the MDA contents detected in transgenic plants were significantly lower than that in the WT (**Figure 3H**).

**FIGURE 4:**
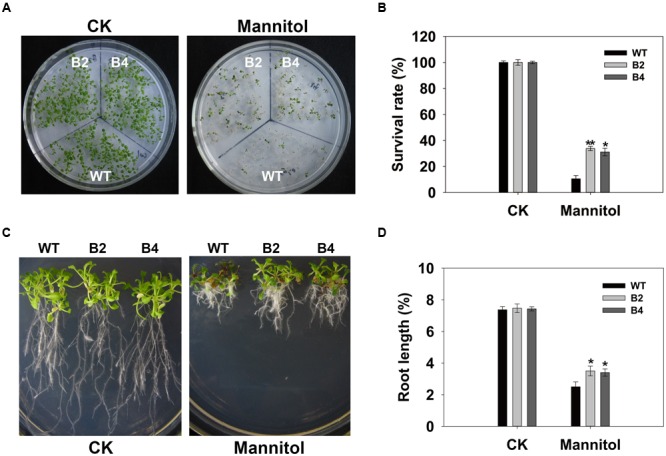
**Overexpression of *OsSGL* enhances osmotic tolerance in transgenic *Arabidopsis*. (A)** Performance of WT and transgenic lines under normal and osmotic stress conditions, respectively. **(B)** Survival rates after osmotic stress. Vernalized seeds of WT and *OsSGL* transgenic plants were sown in triplicate on ½12 MS medium or ½12 MS supplemented with 300 mM mannitol, then monitored for 9 days. Seedlings with green cotyledons were considered survivors. **(C)** After seeds were germinated on ½12 MS medium for 3 days, seedlings were transferred to medium containing 300 mM mannitol and grown vertically for another 12 days. **(D)** Root lengths of WT and transgenic plants after imposition of osmotic stress. Error bars represent SD for 3 independent experiments. ^∗^ and ^∗∗^ indicate significant difference from WT at *P* < 0.05 and <0.01, respectively, by Student’s *t*-test. CK, controls; WT, wild-type *Arabidopsis*; B2 and B4, transgenic Lines 2 and 4, respectively.

Consistent with the results obtained with the transgenic rice, similar osmotic-tolerant phenotypes were observed in transgenic *Arabidopsis* plants hetero-expressing *OsSGL*. Under normal growing conditions, cotyledon opening and the rate of greening did not differ significantly among genotypes (data not shown). However, under osmotic conditions, the transgenic plants had higher survival rates and produced longer roots (**Figures 4A–D**). These results demonstrated that *OsSGL* plays a positive role in osmotic tolerance.

### Up-Regulated Expression of Stress-Responsive Genes in *OsSGL*-Overexpressing Plants

To gain further insights into the mechanism of the improved drought tolerance in *OsSGL*-transgenic plants, RNA-seq analysis was performed with RNA samples extracted from 15-day-old seedlings after osmotic stress. Among the 47 genes that were up-regulated in transgenic plants, 33 were drought-inducible, as had been revealed by previous microarray experiments (GEO accession number GSE6901). In all, 41 target genes showed changes in transcript levels of more than three-fold in *OsSGL*-transgenic plants when compared with the WT (*P*-value < 0.05). These genes encode both functional and regulatory proteins, including stress response proteins, antioxidants, protein kinases, transcription factors, and enzymes involved in metabolism (**Table 1**). Some reactive oxygen species (ROS)-responsive genes also showed higher expression in the transgenic line, including hydroxyacid oxidase (*LOC_Os08g14860*), peroxidase (*LOC_Os07g44499, LOC_Os07g44480*), peroxidase binding protein (*LOC_Os01g73200*), proline-rich protein (*LOC_Os08g15080*), and LEA-related protein (*LOC_Os05g01680*), suggesting that the improved drought tolerance in transgenic plants partially resulted from ROS pathways modulation. We then selected six stress-responsive genes and compared their expression between transgenic and WT plants grown under osmotic stress conditions. qPCR analysis showed an overall pattern of expression similar to that revealed by RNA-seq analysis, confirming the reliability of RNA-seq analysis (Supplementary Figure S3). These data indicated that overexpression of *OsSGL* in rice affects the expression of a set of stress-responsive genes, thereby conferring greater drought tolerance in transgenic plants.

**Table 1:** Genes in *OsSGL*-transgenic plants up-regulated in comparison to wild-type.

Gene ID	log_2_ Ratio	*P*-value	Annotation	Stress response^∗^
LOC_Os07g44499	8.19	8.60E-07	Peroxidase	D, S
LOC_Os08gl4860	6.67	1.25E-29	Hydroxyacid oxidase 1	D
LOC_Os01g52240	6.05	7.61E-36	Type I chlorophyll a/b-binding protein	D
LOC_Os08gl5080	5.58	1.93E-26	Proline rich protein 3	D, S
LOC_Os02g39620	4.95	1.72E-08	Stress-induced protein OZI1	D, S
LOC_Osllg24140	4.53	2.59E-22	Uclacyanin-like protein 35	
LOC_Os07g44480	4.47	2.67E-06	Peroxidase	D, A
LOC_Os01g02010	4.19	4.75E-05	Arabinogalactan protein 12	S, C
LOC_Os08g27850	3.76	7.07E-74	MBF1 transcription factor	
LOC_Os08g28670	3.61	2.19E-15	Bet v I allergen family protein	
LOC_Os01g41710	2.95	5.55E-37	Chlorophyll a-b binding protein 2	D
LOC_Os08g16070	2.91	5.16E-06	Stress response	
LOC_Os07g41350	2.83	6.63E-21	B12D-like protein 1	S, C
LOC_Os06gl6170	2.82	1.10E-06	Protein of unknown DUF231	D
LOC_Os01g58970	2.75	2.57E-10	Cytochrome P450 protein	D, C
LOC_Os06g45940	2.67	5.15E-30	*OsHAKB13*	D, S,C
LOC_Os05gl2280	2.58	9.65E-09	Clumping factor A precursor	
LOC_Osl0g41430	2.46	2.87E-09	Cyclin-U 4;3	D, S,C
LOC_Os01g07700	2.32	2.57E-09	Conserved hypothetical protein	C
LOC_Os07g37240	2.21	1.74E-11	Chlorophyll A-B binding protein	D, S,C
LOC_Os02g44080	2.20	2.57E-09	*OsTIP2*	S, C
LOC_Osl0g31740	2.19	1.60E-06	Glycine-rich cell wall structural protein 2	
LOC_Os06g01210	2.14	1.24E-07	*OsPLAS*	
LOC_Osllg20790	2.12	4.79E-06	Adenylate kinase B	D, S
LOC_Os05g01680	2.09	2.91E-05	LEA related protein	
LOC_Osl0g40700	2.07	1.23E-06	Beta-expansin 6	D, S, C
LOC_Os06g40818	2.07	2.80E-05	Aspartic proteinase	D, S, C
LOC_Os08g01380	2.02	4.79E-05	Ferredoxin I	D, S, C
LOC_Os02g03620	2.00	2.39E-05	RING-HC protein 9	D, S, C
LOC_Os03gl2290	1.99	2.36E-37	*OsGSl;2*	D, S
LOC_Os04g38600	1.94	6.85E-19	*GADPH*	D, S, C
LOC_Os05g47700	1.89	1.94E-11	Lipid transfer protein 2;4	D, S, C
LOC_Os01g73200	1.80	1.26E-39	Peroxidase BP 1	D
LOC_Osl2g36210	1.78	9.13E-19	Similar to MPI.	
LOC_Os07gl5370	1.74	1.59E-09	*OsNRAMPS*	D, S, C
LOC_Os09g25490	1.73	1.17E-12	Cellulose synthase catalytic subunit genes 9	D, S, C
LOC_Os02g07410	1.71	1.05E-09	Glycine cleavage H-protein	D, S
LOC_Os08g05960	1.69	3.48E-10	Defense-responsive gene 10	D, S, C
LOC_Os01gl3690	1.69	4.96E-07	ligA	D, S, C
LOC_Os03g59210	1.68	2.68E-09	Conserved hypothetical protein	D, S
LOC_Os02g51080	1.65	6.26E-06	*LYL1*	D, S, C
LOC_Os08g38170	1.63	2.21E-09	Methyladenine glycosylase	D, S, C
LOC_Os07g36090	1.62	1.01E-06	Ribosomal protein L28	D
LOC_Os05g09440	1.61	4.21E-34	*0sNADP-ME3*	D, S, C
LOC_Os03g08710	1.59	5.25E-08	Thionin 26	
LOC_Os08g01350	1.59	1.57E-05	Transmembrane protein 18	D
LOC_Os03g01700	1.59	1.40E-16	*LSI2*	D, S

*^∗^Genes identified as responsive to ABA (A), cold (C) drought (D), or salt (S) stress are based on microarray profiling data (GEO accession number GSE6901; data sets at http://www.ncbi.nlm.nih.gov/geo/query/acc.cgi?acc=GSE6901).*

### *OsSGL* Alters Root Architecture and Transgenic Rice Plants Produce Deeper Root Systems

**FIGURE 5:**
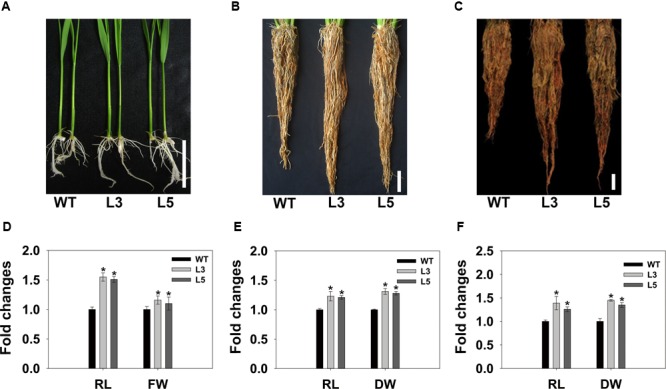
**Comparisons of root growth between WT and transgenic rice plants at seedling, vegetative, and reproductive stages. (A)** Seeds were germinated on ½12 MS medium and plants were grown for 7 days. **(B,C)** Transgenic and WT plants were grouped in individual buckets and grown for 6 and 12 weeks, respectively. **(D–F)** Lengths, fresh weights, or dry weights of transgenic plant roots were normalized to WT plants at seedling, vegetative, and reproductive stage, respectively. Scale bars = 5 cm. Data are means ± SD of 30 roots (10 per growth stage). ^∗^ and ^∗∗^ indicate significant difference from WT at *P* < 0.05 and <0.01, respectively, by Student’s *t*-tests. RL, root length; FW, fresh weight; DW, dry weight. WT, 9311; L3 and L5, transgenic lines L3 and L5, respectively.

Overexpression of *OsSGL* in rice yielded altered phenotypes in root architecture. At the seedling stage, the roots of transgenic plants were approximately 50% longer than those of the WT. Root fresh weights were also significantly higher for those transgenic plants (**Figures 5A,B**, and Supplementary Table S2). To test whether the root enhancement was maintained during subsequent plant development stages, transgenic and WT plants were further cultivated in the same bucket. **Figure 5C** shows that transgenic plants still possessed enlarged root systems. The dry biomasses of the root systems of the two tested transgenic lines were 10.3 and 13.8% higher than that of WT, respectively (**Figure 5D**). In accordance with seedling and vegetative stages, transgenic plants also had larger root systems at the reproductive stage, displaying the increased root dry weight (**Figures 5E,F**).

### *OsSGL* Functions in Regulating Expression of Genes Responsible for Root Development and Hormone Responses

**FIGURE 6:**
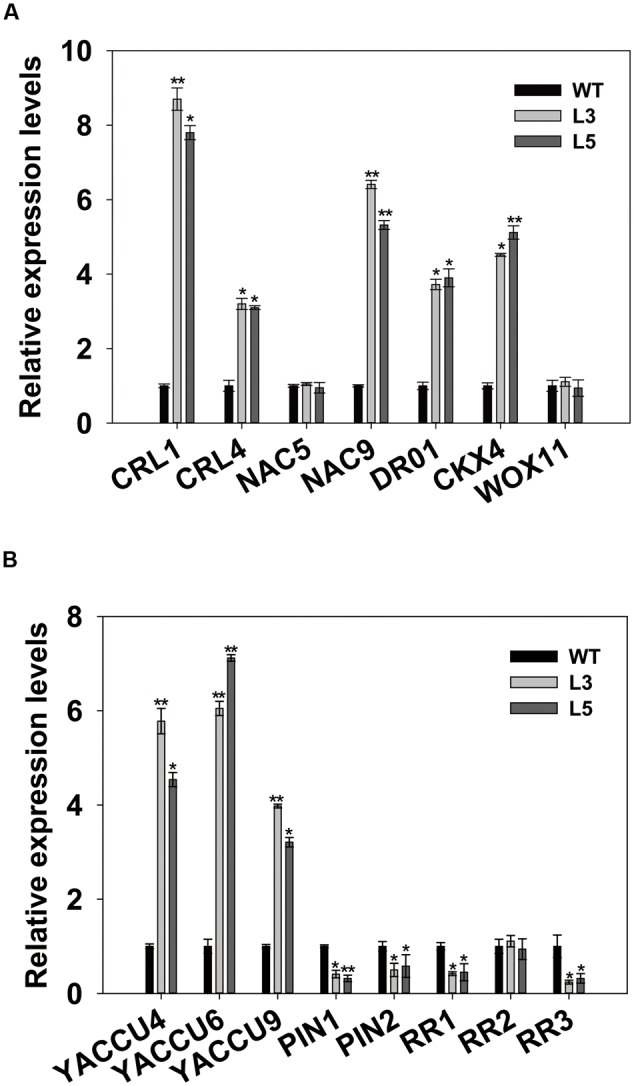
**Expression analyses. (A)** Relative expression levels of root growth-related genes in wild-type (WT) and *OsSGL*-transgenic plants. **(B)** Relative expression levels of auxin- and cytokinin-related genes in WT and transgenic plants. ^∗^ and ^∗∗^ indicate significant difference from WT at *P* < 0.05 and < 0.01, respectively, by Student’s *t*-tests.

The regulatory pathways for root initiation and growth involve *CRL1, CRL4/GNOM1, DR01, CKX4, NAC5, NAC9*, and *WOX11* (Liu et al., 2005; Zhao et al., 2009; Redillas et al., 2012; Jeong et al., 2013; Uga et al., 2013; Gao et al., 2014). To determine whether *OsSGL* regulates these genes, we performed qPCR analysis with root samples from transgenic plants and found that *CRL1, CRL4, DR01, CKX4*, and *NAC9* were highly expressed. This implied that *OsSGL* directly or indirectly influences the expression of those genes. In contrast, transcripts of *NAC5* and *WOX11* were not clearly affected in the transgenic lines (**Figure 6A**).

To determine whether *OsSGL* is also involved in auxin and cytokinin signaling, we analyzed the expression of two auxin transport genes (*PIN1* and *PIN2*), three auxin biosynthesis genes (*YUCCA4, YUCCA6, YUCCA9*), and three cytokinin-responsive Type-A RR genes (*RR1*-*RR3*) in roots from 14-day-old seedlings of WT transgenic rice. While transcript levels of *YUCCA4, YUCCA6*, and *YUCCA9* were elevated in the *OsSGL* transgenic plants, expression of *PIN1, PIN2, RR1*, and *RR3* was repressed, and that of *RR2* was not changed (**Figure 6B**). These results suggested that *OsSGL* regulates genes for root development by modulating the signaling pathways of cytokinin and auxin.

## Discussion

Drought stress, or water deficit, is a primary inhibitor of crop productivity and distribution (Lobell et al., 2014). Global climate change will likely make this situation more serious in the near future (Ahuja et al., 2010). Thus, it is extremely urgent that researchers dissect the functions and mechanisms underlying the activities of genes associated with drought tolerance if we are to develop new drought-tolerant crop varieties through molecular breeding. In this study, the rice gene *OsSGL* was analyzed and evaluated in transgenic rice and *Arabidopsis*. Its expression was induced significantly by osmotic stress, salt, cold, or ABA treatment. This indicated that the gene can respond to multiple environmental cues. To investigate the expression pattern of *OsSGL* orthologs in *Arabidopsis*, we performed a meta-analysis of publicly available microarray data in the Genevestigator database^2^ (Hruz et al., 2008). One DUF1645 member, *AT1G23710*, is induced by salt, cold, drought, ABA, and oxidative stresses (Supplementary Figure S4), which is consistent with the expression pattern we detected for *OsSGL*. Therefore, *AT1G23710* and *OsSGL* may have similar functions in stress responses. The former has not been functionally characterized, and only a few studies have shown that *AT1G23710* expression is altered during cold acclimation, pollen germination, and pollen tube growth (Fowler and Thomashow, 2002; Wang et al., 2008). In the further study, it is fascinating to investigate the possibility of *AT1G23710* involved in the drought tolerance.

Our findings demonstrated that both over- and hetero-expression of *OsSGL* improves tolerance to drought in transgenic plants. Therefore, we can reasonably conclude that this enhanced tolerance might be a result of several beneficial changes at the morphological, physiological, and molecular levels. First, the *OsSGL* transgenic plants produce a more extensive root system that may contribute to greater stress tolerance at the morphological level. The size and architecture of a root system determine plant capacity to access water and nutrients, all factors that can limit growth and yield in many agricultural ecosystems. Therefore, optimization of root system architecture is positively correlated with drought tolerance (Price et al., 1997). Many transcription factors can help improve stress tolerance by altering this architecture (Jeong et al., 2010, 2013; Redillas et al., 2012). We found here that overexpression of *OsSGL* in rice plants led to larger root systems, as evidenced by longer roots and increased biomass. Similar results have been described for transgenic rice plants that over-express *OsEXPA8* (Ma et al., 2013). It is reasonable to assume that longer roots and increased root biomasses may facilitate the absorption of water in deep soil layers, behavior that is considered a drought avoidance strategy (Fukai and Cooper, 1995; Yue et al., 2006). However, in contrast to rice, roots of *OsSGL* transgenic *Arabidopsis* plants were comparable with that of WT under normal conditions (**Figure 4A**). This was probably due to the different genetic backgrounds of monocot and dicot plants.

Phytohormones such as auxin and cytokinin act as necessary regulators of root architecture. Auxin signaling is required for the initiation of rice crown roots, while cytokinin inhibits root growth (Inukai et al., 2005). Auxin–cytokinin crosstalk signaling plays key roles in root development and can coordinately regulate a series of genes (Dello Ioio et al., 2008; Müller and Sheen, 2008). For example, *OsCKX4*, a putative cytokinin oxidase/dehydrogenase gene, mediates crown root development in rice by integrating the interaction between cytokinin and auxin (Gao et al., 2014). Utilizing *ren1-D* mutants of rice, molecular and genetic analyses have revealed that the mutant phenotype is caused by activation of *OsCKX4*. Thus, increased expression of *CRL1* and *CRL4* may be responsible for our observed enhanced-root formation phenotype (**Figure 6A**). Both auxin transport genes, *OsPIN1* and *OsPIN2*, were down-regulated in *OsSGL* transgenic plants, consistent with the results obtained in *ren1-D* mutants (**Figure 6B**). These results were further verified by the fact that auxin biosynthesis genes *YUCCA4, YUCCA6, YUCCA7*, and *YUCCA9* are repressed in the roots of *ren1-D* seedlings when compared with the WT. Overall, these findings suggest that both auxin distribution and auxin biosynthesis are regulated by cytokinin, which is consistent with conclusions from previous studies (Pernisová et al., 2009; Jones et al., 2010). Another auxin- and cytokinin-responsive gene, *WOX11*, is expressed primarily in regions of cell division in both root and shoot meristems (Zhao et al., 2009). In *WOX11*-overexpression lines, *OsRR2*, a Type-A cytokinin-responsive gene, is repressed. Expression of both auxin- and cytokinin-responsive genes is affected by the mutation or overexpression of *WOX11*. Those data suggest that *WOX11* may integrate both auxin and cytokinin signaling to stimulate cell division during the process of crown root development. Our data also showed that overexpression of *OsSGL* either activates or represses some auxin- and cytokinin-responsive genes, which suggests that this gene influences auxin-cytokinin signaling to regulate root growth and development.

A second beneficial change due to *OsSGL* overexpression is the higher accumulation of osmoprotectants and lower MDA contents, which may contribute to the improved stress tolerance in our transgenic plants. Osmotic stress can be a common consequence of drought, high salt, or cold stresses (Apse and Blumwald, 2002; Yamaguchi-Shinozaki and Shinozaki, 2006). In response to osmotic conditions, plants accumulate more osmoprotectants, e.g., proline and soluble sugars, to maintain turgor pressure and to protect enzymes and macro molecules of cells against the damaging effects of ROS (Farooq et al., 2009; Kiani et al., 2007). Proline is an important compatible solute that accumulates in plants exposed to dehydration (Perez-Perez et al., 2007). High levels of free proline allow plants to maintain low water potentials and derive water from the environment. Our *OsSGL* transgenic plants also accumulated more proline and soluble sugars under stress conditions, and they also had less MDA than the WT under osmotic stress. As a product of ROS-stimulated lipid peroxidation, the measuring of MDA contents can be used to evaluate the extent of ROS-mediated injuries in plants (Moore and Roberts, 1998). The higher accumulation of proline in *OsSGL* transgenic plants might contribute somewhat to their lower MDA levels measured under stress conditions. Therefore, the significant increases in amounts of osmoprotectants in our plants may represent one of the main mechanisms underlying improved drought tolerance.

Thirdly, RNA-seq analysis showed a correlation between gene expression profiles and the stress tolerance phenotype observed with our transgenic rice plants. We found it is interesting that some peroxidases were highly expressed in those plants. Peroxidases are involved in many processes, including defense responses to biotic and abiotic stresses. The exposure of plants to unfavorable environmental conditions increases ROS production. Because peroxidases oxidize various substrates that utilize H_2_O_2_ or organic hydroperoxides, they can help in ROS-scavenging. In fact, several plant peroxidases have important roles in the ROS-detoxification process (Miller, 2002; Apel and Hirt, 2004). Two novel pepper peroxidase genes, *CaPO2* and *CaPOD*, are involved in abiotic stress tolerance and pathogen resistance (Choi and Hwang, 2012; Wang et al., 2013). *OsAPX2*, a cytosolic ascorbate peroxidase in rice, is critical for growth and development because it protects seedlings from abiotic stresses through ROS-scavenging (Zhang et al., 2013). In addition to these peroxidases, we also found that some stress-responsive genes were up-regulated in our transgenic plants. Therefore, we might speculate that elevated expression of these anti-oxidative and stress-responsive genes is essential to the development of stress tolerance in *OsSGL* transgenic plants.

In summary, we have demonstrated that over- and hetero-expression of *OsSGL* in rice and *Arabidopsis* plants increases their level of drought tolerance. Some related traits were improved in those transgenic plants, including formation of a more extensive root system, altered transcript levels of stress-responsive genes, and higher accumulations of osmolytes. Additionally, a newly study from our group revealed that OsSGL also controlled the grain length in rice (Wang et al., 2016). These findings provide a good foundation for using *OsSGL* as a target gene for future crop improvement strategies.

## Author Contributions

GX and XX designed the experiments; YC and MW performed the experiments with assistance from XY, GZ, ML, and LH; GX, HZ, and FL analyzed and discussed the results; YC and GX wrote the manuscript.

## Conflict of Interest Statement

The authors declare that the research was conducted in the absence of any commercial or financial relationships that could be construed as a potential conflict of interest.

## Footnotes

1 http://www.ebi.ac.uk/interpro/

2 www.genevestigator.com
