# Supplementary material for: OsSGL, a Novel DUF1645 Domain-Containing Protein, Confers Enhanced Drought Tolerance in Transgenic Rice and Arabidopsis
Source: Front Plant Sci. 2016 Dec 27;7:2001. doi: 10.3389/fpls.2016.02001 (PMC5186801; doi:10.3389/fpls.2016.02001)
Supplement: Supplementary file 1 [file Data_Sheet_1.pdf]

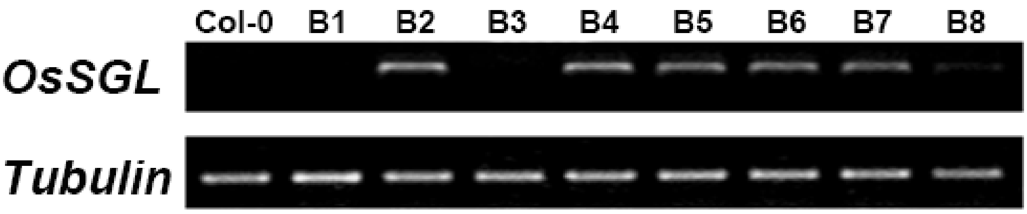

FIGURE S1 Analysis of *OsSGL* in transgenic Arabidopsis plants. Transcript levels of *OsSGL* in the transgenic Arabidopsis lines, as revealed by semi RT-PCR analysis. The t*ubulin* gene was used as internal control. Col-0, wild type; B1-B8, transgenic line1-line8.

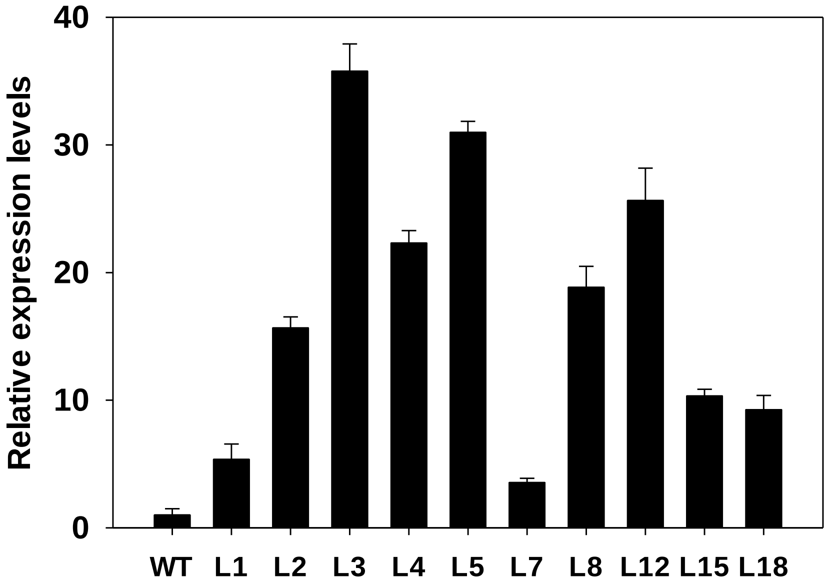

FIGURE S2 qPCR analysis of *OsSGL* expression in transgenic rice lines and wild type plants. Total RNAs were extracted from different transgenic lines of T_3 generation and wild type plants. Error bars represent SD for three independent

experiments. WT, wild type; L1-L18, transgenic lines.

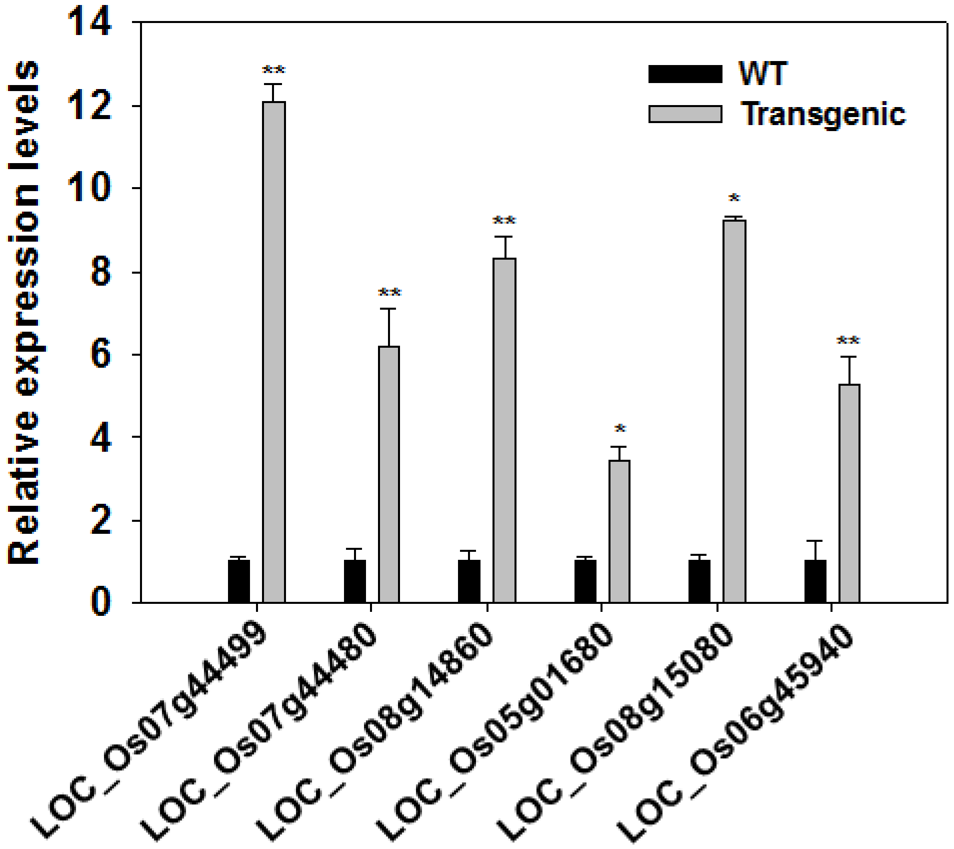

FIGURE S3 Relative expression levels of some highly up-regulated genes in transgenic rice plants selected based on RNA-Seq analysis. qPCR was used to analyze the expression levels. The data represent the mean ± SE (n = 3). * indicate a significant difference from that of WT at * $P < 0.05$ and ** $P < 0.01$, by Student's t-test.

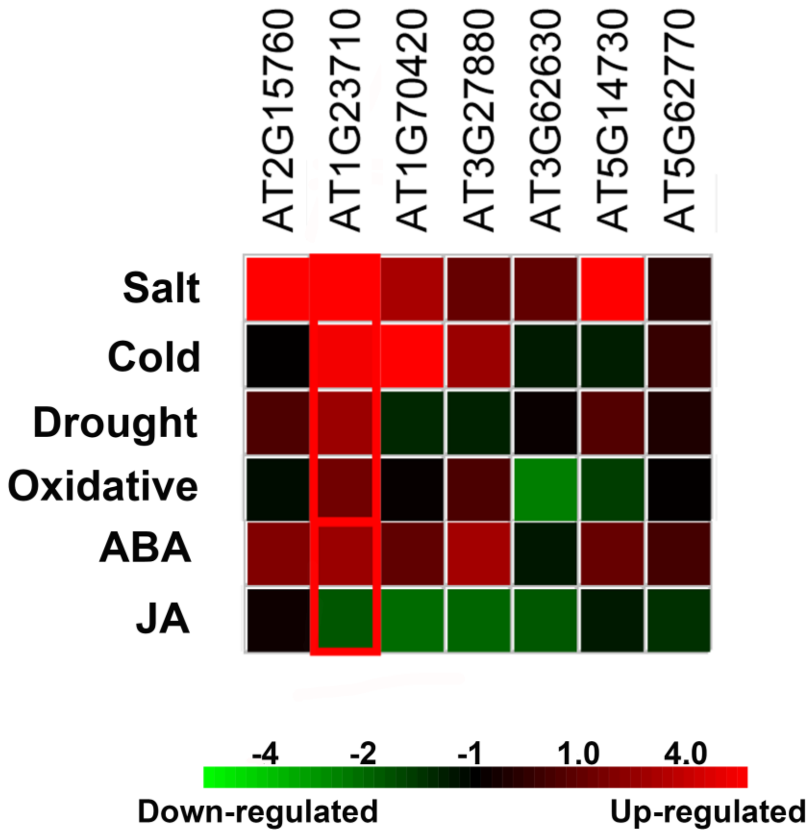

FIGURE S4 Expression analysis of DUF1645 orthologous genes in Arabidopsis under stresses and hormone treatment. The analysis was performed using a meta-analysis of selected publicly available microarray data sets lodged with the Genevestigator database (www.genevestigator.com). Data of salt, drought, cold, and oxidative stresses were obtained from experiment ID-AT00120; ABA and JA were obtained from experiments AT-00637 and AT-00683, respectively.

TABLE S1 List of primers used in this study.

**Primer name**	**Primer sequence (5' to 3')**	**Purpose**
OsSGL-F	CCATGGAAGACGACGTGATGCCACCCAGC	Overexpression
OsSGL-R	AACTAATAGGCGGTGTGGTGTTGCG	
OsSGL-RT-F	CCGCCATCATCCAAACTGA	qRT-PCR and semi RT-PCR
OsSGL-RT-R	GGTGACCACGCCCTTCTTC	
HptII-F	ACCTGCCTGAAACCGAACTG	Check transformants
HptII-R	CTGCTCCATACAAGCCAACC	
AtTubulin-F	CGTGGATCACAGCAATACAGAGCC	qRT-PCR and semi RT-PCR
AtTubulin-R	CCTCCTGCACTTCCACTTCGTCTTC	
OsActin-F	GATGACCCAGATCATGTTTG	
OsActin-R	GATGACCCAGATCATGTTTG	
OsSGL-GFP-F	GGATCCCTAAGAATCCAATCCACTCCACTC	Subcellular localization
OsSGL-GFP-R	CCATGGCGTTATAGGCGGTGTGGTGTTGC	
LOC_Os07g44499-F	CCCGCCAACAACCTATCCT	
LOC_Os07g44499-R	TTCTTGACGGTGTCCTTCACA	
LOC_Os07g44480-F	GCTCACATTGCTGGCTCTGG	
LOC_Os07g44480-R	GGCTTGGTGGAAGATAGGTTGT	
LOC_Os08g14860-F	CAGCGGCTCAAGCGCACCAT	
LOC_Os08g14860-R	TCCCATCGCCGTCGTTCTCG	
LOC_Os05g01680-F	ACGAAGCAGGGAGGAGTGGG	
LOC_Os05g01680-R	GCAGAAGGTCTTGCAGGTGGG	

LOC_Os08g15080-F	TCAACTTCAAGGGCGACCAG	qRT-PCR
LOC_Os08g15080-R	TGTGCAGCGCCGTCTTGGTC	
LOC_Os06g45940-F	ATGAAGGTCGTCGAGTTCC	
LOC_Os06g45940-R	GTCAAAGGCGTGCTGATT	
NAC9-F	CTGAGCTACGACGATATCCA	
NAC9-R	GAAGAGCGACGAGTAGAAGT	
CKX4-F	GACCGACTACCTCCATCTCACA	
CKX4-R	GGTTGACATTGCTGACCTGC	
DR01-F	GCAAGAAGCAAATCGGTTTCC	
DR01-R	GAATTCATCCTTTCGACAATCTGA	
YUUCA4-F	TGGCCTGTACGCTGTTGGTTTCT	
YUUCA4-R	CCATTCCATGTCAGTTGTTCTCA	
YUUCA6-F	GTCAGGGCTCTACTCTGTTGGCT	
YUUCA6-R	ATCTTGTGGTGGCGGTTTGGT	
YUUCA7-F	AGGAGGCGACGAAGCCAAC	
YUUCA7-R	TCAACAACGAATTTAACCAAGGG	
RR1-F	GGCGAAACTGGGCAATAG	qRT-PCR
RR1-R	GCCTCCACAAGGAGATGATACTG	
RR2-F	ATTTTGCTGAGAGAGAAAAGAGTAG	
RR2-R	ACGACACCAGATGCCCACTC	
RR3-F	CGCAGCTCCAAATATCGAGTTAC	
RR3-R	CACATTCCGATCCAGGCTGAG	

WOX11-F	CCCTTGTCCGTTGGATCAGA	qRT-PCR
WOX11-R	CCATGCGTACGTGCAGCATT	
CRL1-F	ATGACGGGATTTGGATCGC	
CRL1-R	CTTGCTCGTGGCAGAAGTAT	
CRL4-F	GGATTGGGAATGCTACTTCG	
CRL4-R	CCTTCTTTGGGTCTCTGTTG	
PIN1-F	TCTCGCTCGGACATCTACTCCC	
PIN1-R	AGTCCTCCCTGTCCTTCGCTC	
PIN2-F	GCCGCTCCTCTCCTTCCACTT	
PIN2-R	CATGAGGGTGTACCAGATGA	
NAC5-F	CCGCAAGCTCTCCAAGTCCT	
NAC5-R	TCACCGTGTCGTACCTCTC	

Table S2 Characteristics of one-week old seedlings of WT and transgenic line L3 (Mean ±SD)

**Trait**	**WT**	**L3**
Shoot length (cm)	9.281 ± 0.681	9.318 ± 0.561
Adventitious root length (cm)[a]	2.013 ± 0.362	2.703 ± 0.514**
Lateral root length (cm)[b]	0.152 ± 0.011	0.226 ± 0.017**

a The average length of the 5 longest adventitious roots.

b The average length of the 15 longest lateral roots on each primary root.

** $p<0.01$ compared with WT (Student's *t* test)
